# The A, B, Cs of Herpesvirus Capsids

**DOI:** 10.3390/v7030899

**Published:** 2015-02-26

**Authors:** Ritesh Tandon, Edward S. Mocarski, James F. Conway

**Affiliations:** 1 Department of Microbiology and Immunology, University of Mississippi Medical Center, 2500 North State Street, Jackson, MS 39216, USA; 2 Department of Microbiology and Immunology, Emory University School of Medicine, 1462 Clifton Road, Atlanta, GA 30322, USA; E-Mail: mocarski@emory.edu; 3 Department of Structural Biology, University of Pittsburgh School of Medicine, Biomedical Science Tower 3, 3501 5th Avenue, Pittsburgh, PA 15260, USA; E-Mail: jxc100@pitt.edu

**Keywords:** CMV, HSV, envelopment, nucleocapsid, trafficking, assembly

## Abstract

Assembly of herpesvirus nucleocapsids shares significant similarities with the assembly of tailed dsDNA bacteriophages; however, important differences exist. A unique feature of herpesviruses is the presence of different mature capsid forms in the host cell nucleus during infection. These capsid forms, referred to as A-, B-, and C-capsids, represent empty capsids, scaffold containing capsids and viral DNA containing capsids, respectively. The C-capsids are the closest in form to those encapsidated into mature virions and are considered precursors to infectious virus. The evidence supporting A- and B-capsids as either abortive forms or assembly intermediates has been lacking. Interaction of specific capsid forms with viral tegument proteins has been proposed to be a mechanism for quality control at the point of nuclear egress of mature particles. Here, we will review the available literature on these capsid forms and present data to debate whether A- and B-capsids play an important or an extraneous role in the herpesvirus life cycle.

## 1. Introduction

Herpesvirus nucleocapsid assembly follows a pathway similar to the assembly pathway of tailed dsDNA bacteriophages [1,2,3]. Most notable among these similarities are the presence of bacteriophage HK97-like floor domain in major capsid protein (MCP) of herpesviruses and the similarity in their terminases [4,5] and ATPases [6]. Moreover, both tailed bacteriophages and herpesviruses use powerful ATP-driven molecular motors to translocate viral DNA into a preformed capsid shell [7,8] and the pressure-driven DNA ejection is also conserved in both groups [9]. In both cases, the primary capsid, known as procapsid, is assembled upon virus-encoded scaffolding proteins, which are later digested by the action of the viral protease, clearing the way for viral DNA packaging [10,11]. Assembly in both groups results in the incorporation of a portal complex at a unique capsid vertex [12] although Cardone *et al.* have reported that in herpesvirus, the portal is mounted on the outer surface of the capsid floor layer, with its narrow end pointing outwards, which is different from that of known phage portals in that the bulk of its mass lies outside, not inside, the floor [13]. Herpesviruses encode vastly more virion-associated proteins compared to bacteriophages, and their assembly takes place in both nuclear and cytoplasmic compartments of infected cells [11]. Herpesviruses also require additional factors to maintain nucleocapsid stability, including the triplex molecule, a unique and essential component of herpesvirus capsids [14,15,16], as well as tegument proteins that form a layer between envelope and nucleocapsid [11,17,18]. In herpesviruses, nucleocapsid stability, and especially the retention of pentons, is regulated by the formation of disulfide bonds [19,20] whereas, covalently joined subunits that loop through each other, contribute to capsid stability in bacteriophages [21]. Maturation of herpesvirus nucleocapsids into infectious virions requires nuclear tegumentaion, nuclear egress, cytoplasmic tegumentation and envelopment [10,11,18,22]. The virus escapes by crossing the inner and outer nuclear membranes and undergoes a primary envelopment and de-envelopment during this process [22,23]. Herpesviruses are also unique in the sense that three major mature capsid forms are normally detected in the infected host cell nucleus. These capsid forms, namely A, B, and C, represent empty capsids, scaffold containing capsids and viral DNA containing capsids, respectively [10,11,17,18,24,25]. There is little doubt that C-capsids mature into infectious virions; however, the role of A- and B-capsids in the life cycle of herepesviruses remains unclear. These forms may represent assembly intermediates or abortive capsid forms generated as a result of unsuccessful proteolytic digestion of capsid scaffold or a failed attempt to package viral DNA. B-capsids contain cleaved assembly precursor (AP) suggesting that the capsid is either preparing to package or that the scaffold was not removed in time to allow for DNA packaging. The genome packaging machinery works in fractions of a second [26,27,28,29,30]. Therefore, it is possible that the capsids shape and stability is maintained due to the rapid packaging of viral genome. All three capsid forms are present in the nucleus, as well as in the cytoplasmic compartments and also undergo primary and secondary envelopments; however, C-capsids predominate in the cytoplasm. When released from the cell, A- and B-capsids appear to constitute a population called noninfectious enveloped particles (NIEPs) [10,11,31].

In order to study the role of these capsid forms in human cytomegalovirus (HCMV) assembly and maturation, we used a benzimidazole ribonucleoside [2-bromo-5,6-dichloro-1-(β-d-ribofuranosyl) benzimidizole (BDCRB)], a virus encapsidation inhibitor that inhibits human cytomegalovirus (HCMV) replication without inhibiting viral DNA synthesis [32]. The polygenomic concatemeric HCMV DNA does not mature to unit genome length in the presence of BDCRB, resulting in an encapsidation block [32]. We show excessive accumulation of the B-capsids but not the A-capsids close to nuclear envelope during BDCRB mediated encapsidation block indicating that B-capsids represent putative virus assembly intermediates and raising important questions about the role of different capsid forms in HCMV life cycle.

## 2. Materials and Methods

### 2.1. Cells

Primary human foreskin-derived fibroblasts (HF) cells were cultured in Dulbecco’s modified Eagle’s medium (Invitrogen Corporation, Carlsbad, CA, USA) containing 4.5 g/mL glucose, 10% fetal bovine serum (S1245OH; Atlanta Biologicals, Lawrenceville, GA, USA), 1 mM sodium pyruvate, 2 mM l-glutamine, and 100 U/mL penicillin-streptomycin (Cellgro, Manassas, VA, USA) at 37 °C with 5% CO_2_. HFs between passages 5 and 15 were used for infections. The cell culture medium was replaced every other day.

### 2.2. Chemical Inhibition

2-bromo-5,6-dichloro-1-(β-d-ribofuranosyl) benzimidizole (BDCRB; 20 µM, a gift from John Drach, University of Michigan, Ann Arbor, MI, USA) was added 1 h post infection (hpi) and maintained up to 7 days post infection (dpi). In some experiments, labeled as BDCRB block-release, BDCRB added at 1 h post infection (hpi) was maintained until 4 dpi, when cultures were washed four times with medium then further incubated in drug-free medium for 3 more days before fixing for processing at 7 dpi.

### 2.3. Transmission Electron Microscopy

Samples for transmission electron microscopy (TEM) were prepared by infecting HFs with the Towne strain of HCMV at an MOI of 3.0. In each case, cells were fixed at endpoint in 2.5% glutaraldehyde in 0.1 M cacodylate buffer (pH 7.2) for 2 h at room temperature. Cells were then washed with the same buffer and postfixed with buffered 1.0% osmium tetroxide at room temperature for 1 h. Following several washes with 0.1 M cacodylate buffer, cells were dehydrated with ethanol, infiltrated, and embedded in Eponate 12 resin (Ted Pella Inc., Redding, CA, USA). Cell culture plates were cracked with a hammer to release the resin after it had solidified, and ultrathin sections (60 to 70 nm) of monolayer cells were cut and counterstained using uranyl acetate and lead citrate. Examination of ultrathin sections was carried out on a Hitachi H-7500 TEM (Hitachi High-Technologies Corporation, Tokyo, Japan) operated at 75 kV, and images were captured using a Gatan BioScan (Pleasanton, CA, USA) CCD camera. The images were acquired and analyzed with Gatan Digital Micrograph software (Version 2.0, Pleasanton, CA, USA, 2014). Average number of particles from 6–9 different micrographs spanning at least 3 different infected cells for each sample were identified based on their established characteristics [17] and enumerated before peforming statistical analysis (Version 6, GraphPad Software, La Jolla, CA, USA, www.graphpad.com). Statistical significance was determined using the Holm-Sidak method, with alpha = 5.000%. Each row was analyzed individually, without assuming a consistent scattered distribution.

### 2.4. Virus and Capsid Purification

HCMV capsids were purified using established protocols for herpesvirus capsid purification [33,34,35] with some modifications. Briefly, HF monolayers were infected with the Towne strain of HCMV (MOI of 5), and harvested at 4 to 5 days postinfection. Cell pellets were washed in 1XPBS and incubated in hypotonic buffer (20 mM Tris-HCl, pH 7.5) for 20 min to swell before adding Triton X-100 (1.5% final concentration) to lyse the cells for 30 min. Nuclei were spun down at 2000 *g* for 10 min, resuspended in TNE (500 mM NaCl, 10 mM Tris-HCl, 1 mM EDTA, pH 8.0), sonicated (Sonicator 3000, Misonix Incorporated, Farmingdale, NY, USA) for 10 s and spun at 14000 rpm for 30 s in a microcentrifuge. The above step was repeated once and combined supernatants were loaded on 20%–50% discontinuous sucrose gradient in TNE before centrifugation (SW-41 rotor, L-80 ultracentrifuge, 24,000 rpm, 1 h, Beckman Coulter, Inc., Indianapolis, IN, USA). Capsids were observed as visible light-scattering bands with A-capsids forming a thin upper band around 30% gradient and B-capsids forming a thick lower band around 35% gradient; the C-capsid band was faint. The B-capsids were harvested by puncturing the side of the centrifuge tube with a 23 G needle and were washed once in TNE before concentrating by centrifugation (SW-41 rotor, Beckman L-80 ultracentrifuge, 24,000 rpm, 1 h). Identity and purity of harvested capsids was confirmed by transmission electron microscopy.

### 2.5. Cryo-Electron Microscopy

Three microliters of purified capsids were pipetted onto a freshly glow-discharged Quantifoil R2/1 grid (Quantifoil Micro Tools GmbH, Jena, Germany) and mounted in an FEI Vitrobot Mk III (FEI, Hillsboro, OR, USA) for blotting and plunge-freezing into a 60:40 mix of liquid ethane/propane. Grids were transferred onto a Gatan 626 cryoholder and mounted into an FEI TF20 cryo-electron microscope (Hillsboro, OR, USA) maintaining liquid nitrogen temperature throughout. The microscope was operated at 200 kV and the sample was imaged using standard low-dose conditions at a magnification of 50,000× on a Gatan UltraScan 4000 CCD camera (Pleasanton, CA, USA) with a post-column magnification of 1.4×. The final pixel size corresponded to 2.1 Å at the sample. Image reconstruction was performed with the AUTO3DEM package [36] and the resolution estimated for the A- and B-capsid maps were 21 Å and 19 Å, respectively, according to the Fourier shell correlation limit of 0.5.

## 3. Results

**Figure 1:**
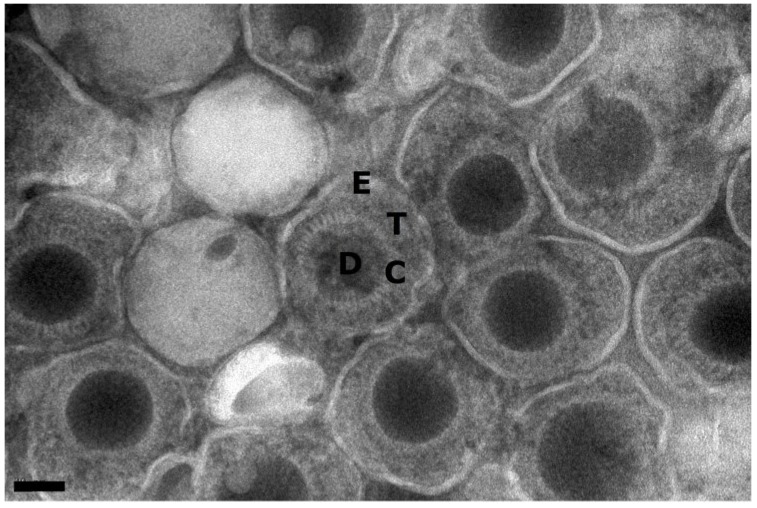
Transmission electron micrograph of a negatively stained preparation of purified extracellular HCMV (Towne) virions. The virion in the center is labeled for the genomic DNA (D), Capsid (C), Tegument (T), and envelope (E) layers. The dense bodies, composed of tegument proteins but no nucleocapsid, can be seen to the left of the labeled virion. Scale 40 nm.

**Figure 2:**
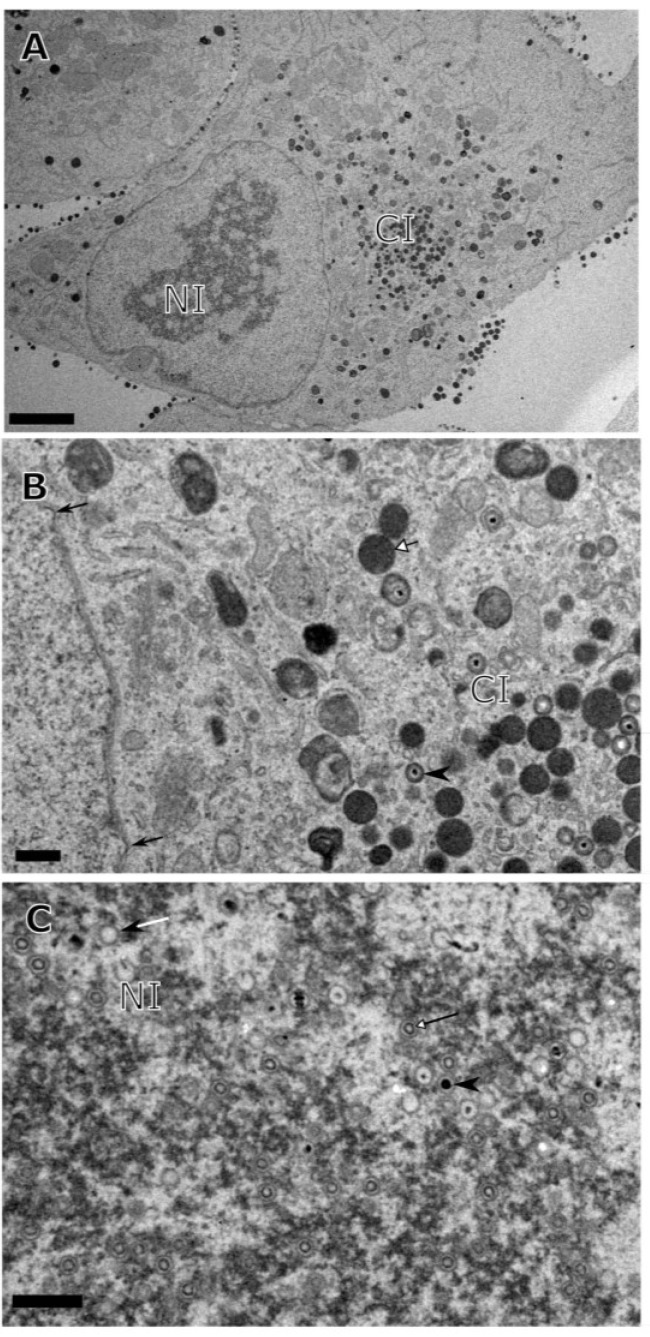
Transmission electron micrograph of HF cells infected with HCMV (Towne) and fixed for processing at 7 days post infection. (**A**) A single infected cell showing nuclear inclusion (NI), as well as cytoplasmic inclusion (CI); (**B**) Interface of nucleus and cytoplasm illustrating nuclear budding events (black arrows), mature virions (arrowheads) and dense bodies (open head arrows); (**C**) Infected cell nucleus showing the distribution of A- (white tailed arrows), B- (black tailed arrows) and C- capsids (arrowheads). Scale: 4 µm (**A**), 0.5 µm (**B**), 0.4 µm (**C**).

Examination of mature cytoplasmic or extracellular HCMV virions reveals four distinct layers: viral DNA (D), capsid (C), tegument (T), and envelope (E), which can all be distinguished in transmission electron micrographs of negatively stained samples (Figure 1). During productive HCMV infection, capsids are visible by electron microscopy as early as two days post infection in the nucleus of infected cells. Three populations of capsids can be identified: empty A-capsids, scaffold containing B-capsids and viral genomic DNA containing C-capsids (Figure 2C). These capsid populations are unevenly distributed in the nucleoplasm. Typically, B-capsids make up the majority (~80%), followed by C-capsids (15%) and then A-capsids (5%) [17]. Most capsid forms are found to be tightly associated with the darker stained areas of the nucleus, termed nuclear inclusions (NI), which are nuclear replication compartments (NRC) that contain replicating virus DNA [18] (Figure 2C). These capsids do not accumulate in the vicinity of nuclear envelope suggesting a rather rapid transport of capsids from the NRC to the inner nuclear membrane (INM) for nuclear egress. The presence of NI is a characteristic of cells infected by any herpesvirus although cytoplasmic inclusions (CI) appear to be unique to cytomegalovirus infected cells (Figure 2) [18,37,38,39,40]. While NI are associated with the centers of viral DNA replication in the nucleus, CI are associated with virus maturation events occurring in the cytoplasm [41,42,43,44]. Also, the changes in the size and morphology of the nucleus that are observed in HCMV infected cells are distinct from the changes in cells infected with other herpesviruses [11]. Capsids are assembled in the nucleus from viral proteins that are imported from the cytoplasm and the assembly can take place in the absence of viral DNA as evidenced by HSV capsid formation in a virus-free and in an *in vitro* cell free system [45,46]. In HCMV, capsids assemble from major capsid protein (MCP/pUL86), triplex monomer (TRI1/pUL46, also called minor capsid binding protein (mcBP)), triplex dimer (TRI2/pUL85, also called minor capsid protein (mCP)) and smallest capsid protein (SCP/pUL48A) with help from a pUL80-based scaffold that translocates MCP into the nucleus and organizes the capsid shell before being cleaved by the viral maturational protease (pUL80a) [17,18]. Preformed capsids process the scaffold as viral DNA is encapsidated by the TER complex (UL89, UL56, UL51) through a PORT (UL104) [18]. A putative capsid vertex specific complex (CVSC) constituted of pUL77 and pUL93 is believed to bind onto nucleocapsid pentamers, with pUL95, pUL52, pUL32, and pUL96 added later on for nucleocapsid stabilization [18]. Staining for major capsid protein (MCP) in infected cells demonstrates accumulation of this protein in NI but not anywhere else in the nucleus [40,47]. It is not clear how capsid proteins and capsids themselves are restricted to the NI in the absence of a physical barrier. The A-, B-, and C-capsids are stable enough to be readily isolated from the nuclei of HSV-1-infected cells [25]. For HCMV, although all three types of capsids can be harvested, the C-capsids lose their stability very quickly after harvest (personal experience and [48]).

**Figure 3:**
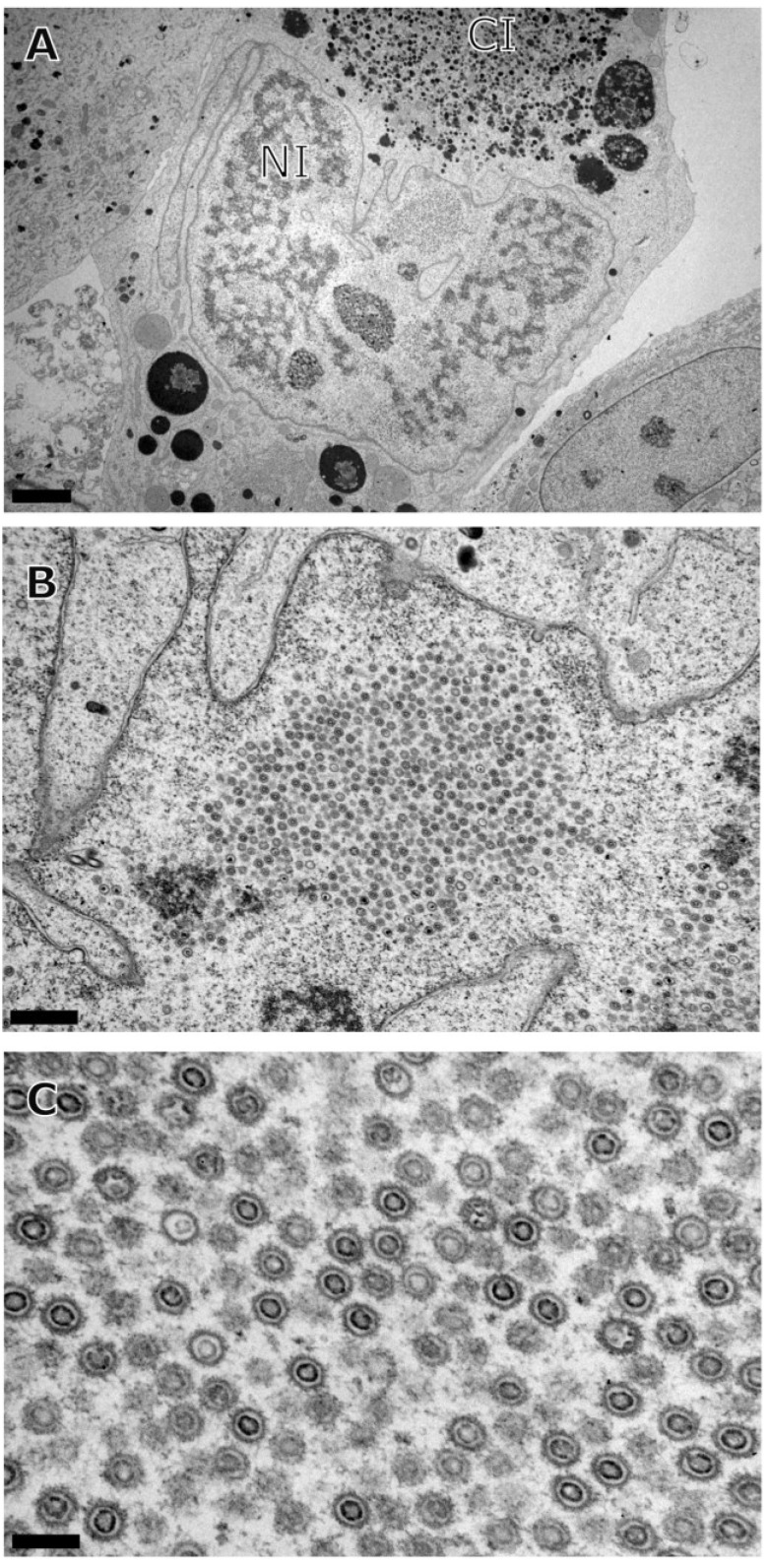
Transmission electron micrograph of HF cells infected with HCMV (Towne) in the presence of BDCRB and fixed for processing at 7 days post infection (**A**) A single infected cell showing nuclear inclusion (NI) as well as cytoplasmic inclusion (CI); (**B**) A part of the nucleus demonstrating excessive accumulation of B-capsids in the center of the micrograph; (**C**) A higher magnification image of the mostly B-capsids accumulating in the nucleus. Scale: 4 µm (**A**), 1 µm (**B**), 0.4 µm (**C**).

To investigate the role of different types of capsids in HCMV maturation, we blocked the encapsidation of the HCMV genome using BDCRB. BDCRB blocks the processing and maturation of viral DNA, specifically inhibiting the insertion of the genomic DNA into the capsid by preventing a necessary interaction of large subunit of terminase with the portal [49,50]. This block was maintained for prolonged periods (seven days post infection) with no obvious toxicity to cells. This allowed for the accumulation of intermediate or abortive forms of capsids in the nucleus. Examination of infected cells by transmission electron microscopy revealed mostly B-capsid forms in the nucleus, confirming a block prior to encapsidation but after capsid assembly (Figure 3). A-capsids were observed in the nucleus but their numbers were negligible compared to the B-capsids. Numerous B-capsids were seen close to the INM, away from NRC. Close examination of these particles revealed that these were identical to the B-capsids that were associated with the NRC. Interestingly, A-capsids that were present close to the NRC did not move to the areas away from NRC where B-capsids accumulated. Examination of the cytoplasmic areas close to the nucleus revealed cytoplasmic inclusions filled with dense bodies (DB), the tegument containing capsid-less structures that are a previously described cytoplasmic maturation byproduct of replication [11,17,18]. Nuclear budding was intact and some B-capsids were observed in the cytoplasm (Figure 3B).

**Figure 4:**
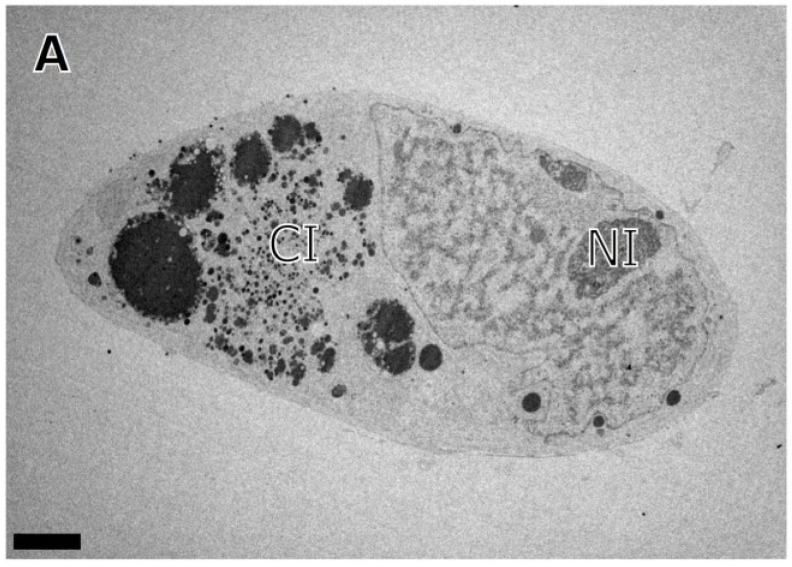

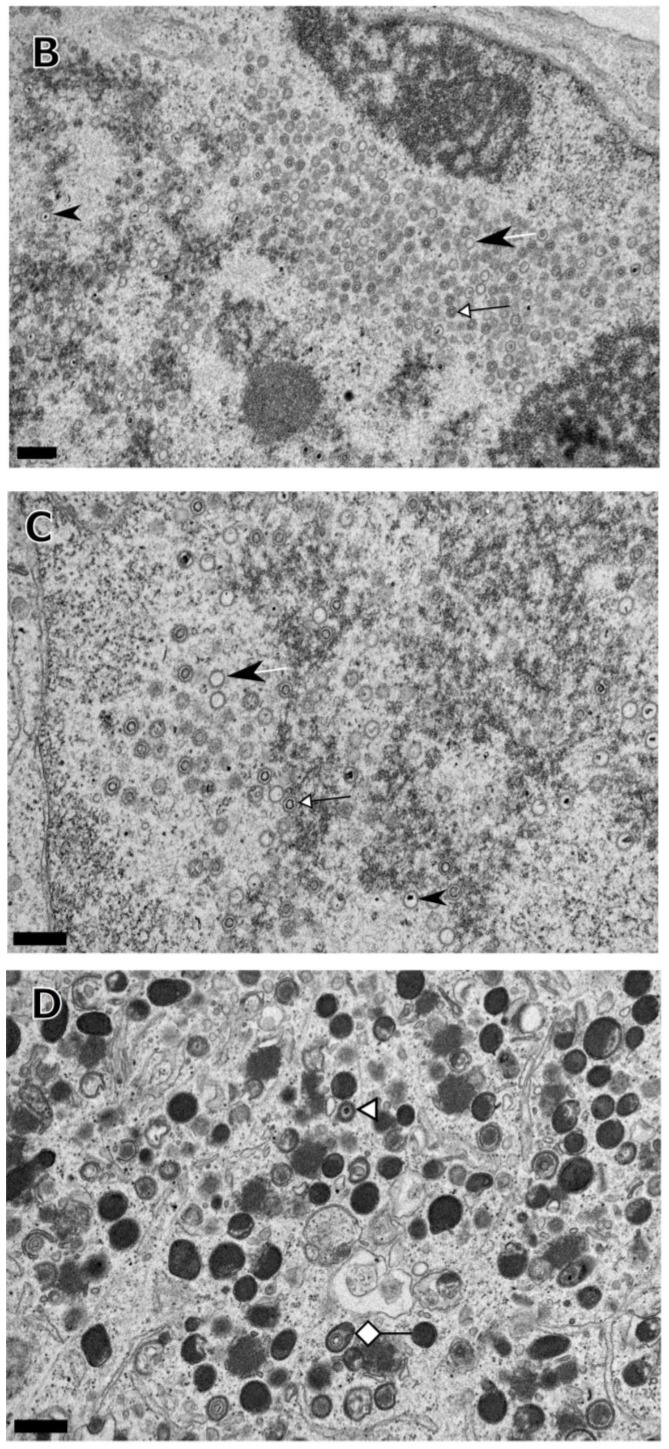
Transmission electron micrograph of HF cells infected with HCMV (Towne) and incubated in the presence of BDCRB for 4 days, the drug was then washed out and cells were incubated further for 3 days in the absence of BDCRB before processing. (**A**) A single infected cell showing nuclear inclusion (NI) as well as cytoplasmic inclusion (CI); (**B**) A part of the nucleus demonstrating mostly B-capsids on the right half of the image and a mix of A-, B- and C-capsids on the left half of the image; (**C**) A higher magnification image of the part of the nucleus containing a mixed population of capsids; (**D**) A part of the cytoplasmic inclusion (CI) showing enveloped virus particles. Scale: 4 µm (**A**), 0.5 µm (**B**, **C**, **D**). A- (white tailed arrows), B- (black tailed arrows), C-capsids (arrowheads), enveloped C-capsids (triangular arrowhead) and enveloped B-capsids (diamondhead).

**Figure 5:**
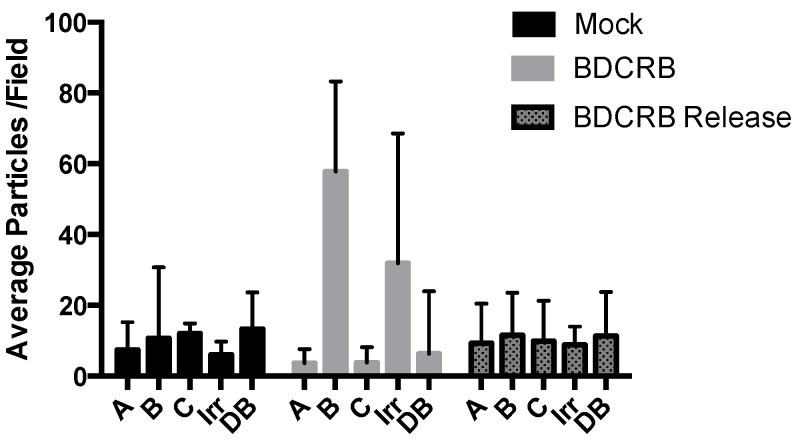
Distribution of different virus capsids and particle types in TEM micrographs of HF that were infected with HCMV (Towne) and were either mock-treated, BDCRB-treated or BDCRB-treated and released as described in materials and methods. Average number of particles from 6–9 different micrographs spanning at least 3 different infected cells for each sample were identified based on their established characteristics [17] and enumerated before peforming statistical analysis (GraphPad Software). Differences in the number of B-capsids between mock-treated and BDCRB treated samples were statistically significant (*p* < 0.05). Variations in the number of particles per field led to the higher than usual error bars. A: A-capsids, B: B-capsids, C: C-capsids, Irr: Irregular shaped particles, DB: Dense bodies.

**Figure 6:**
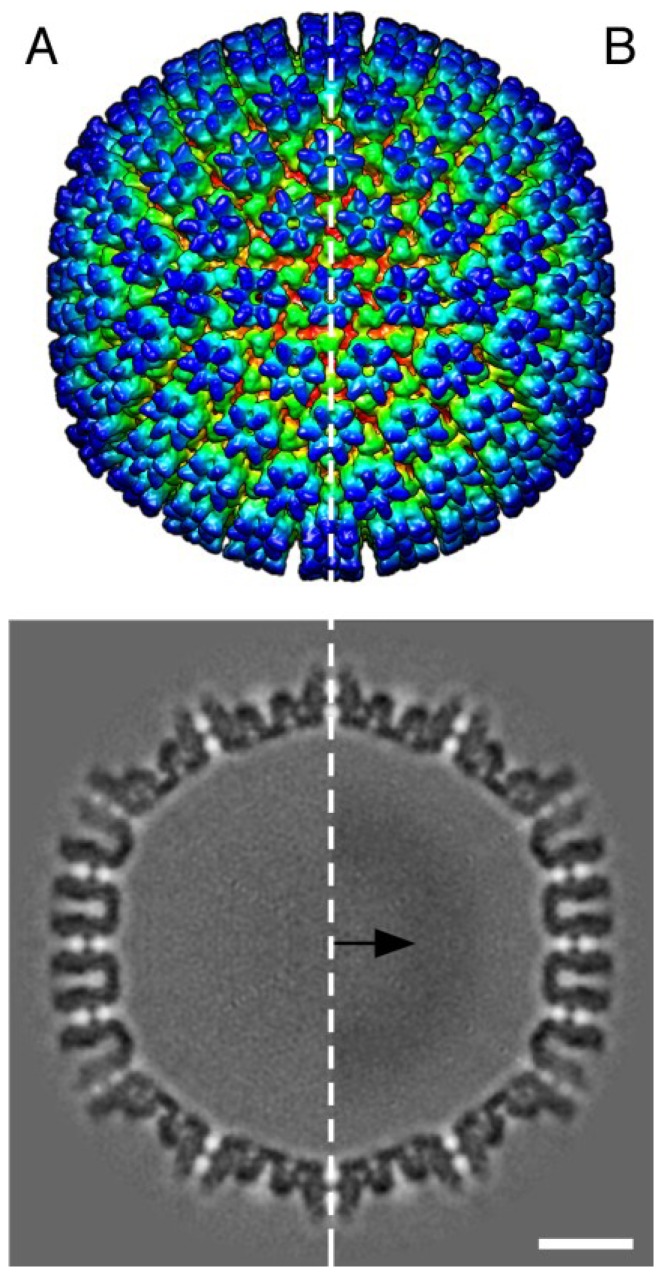
Cryo-EM reconstructions of the HCMV A- ((**A**), left panel) and B-capsids ((**B**), right panel). These two capsid types appear to be identical at this resolution. Surface views are shown on the top panels and sections are shown below. The arrow indicates the B-capsid “core” of poorly organized scaffold remnants that are icosahedrally blurred into the dark ring. A-capsids lack this internal density. Scale 20 nm.

To investigate the fate of B-capsids that accumulated near INM during BDCRB block, we repeated the experiments with the difference that the BDCRB-block was released at four days post infection and the infection was followed for three days post BDCRB-release. A distribution of A-, B-, and C-capsids close to NRC was observed (Figure 4B). The population of B-capsids that had accumulated away from NRC and close to INM in BDCRB-blocked cell was still present in released cells, suggesting that these capsid forms do not proceed to genome encapsidation once the BDCRB block is released. Instead, a number of newly formed C-capsids could be observed close to the NRC (Figure 4C). Examination of CI revealed the presence of enveloped B- as well as C-capsid forms in the cytoplasm (Figure 4D). Distribution of different virus capsids and particle types in TEM micrographs of mock-treated, BDCRB-treated or BDCRB-treated and released infected cells were determined by enumeration of these particle types in six to nine different micrographs spanning at least three different infected cells for each sample (Figure 5) [17]. Differences in the number of B-capsids per field of mock-treated and BDCRB treated samples were statistically significant (*p* < 0.05). More than 85% of capsids present in the nucleus of BDCRB-treated cells were B-capsids compared to only about 50% in mock-treated or BDCRB-released cells at this late time post infection (7 dpi) (Table 1). This is not surprising considering that the active quality control mechanisms at the nuclear membrane selectively allow for the budding of C-capsids and not the A- and B-capsids, leading to the build-up of B-capsids in the nucleus during BDCRB block. That’s also why we don’t see many cytoplasmic or surface-associated capsids in BDCRB treated cells. Table 1 also lists the absolute counts of nuclear capsids per nucleus examined and it indicates that the only the ratios of capsids and not the total number of capsids change significantly during BDCRB block compared to mock-treated cells, addressing the concerns about the overproduction of B-capsids during BDCRB block. We harvested A- and B-capsids from the nucleus of HCMV infected fibroblasts and purified them on a sucrose gradient. Cryo-EM imaging and reconstructions of these capsids revealed structures that were closely similar to each other (Figure 6) and only differed from the published virion structure in terms of the density of tegument proteins that are present on the surface of virions [51] but not on the nuclear capsids. The A- and B-capsid structures were similar to those reported for simian cytomegalovirus [48] and simian rhadinovirus [25].

**Table 1:** Distribution of nuclear capsid types in mock-treated, BDCRB-treated and BDCRB released infected cells. Average number of particles from 6–9 different micrographs spanning at least 3 different infected cell for each sample were identified based on their established characteristics [17].

Type of nuclear capsid	Mock-treated	BDCRB-treated	BDCRB release
A (% of total)	27.30	10.75	25.00
B (% of total)	52.90	85.05	50.80
C (% of total)	19.83	4.20	23.40
Average capsids per nucleus	60.50	71.33	41.33

## 4. Discussion

The presence of three distinct mature forms of capsids in the nuclei of herpesvirus-infected cells poses an interesting conundrum as to the role of these capsid forms in the herpesvirus life cycle. Are these forms merely the byproduct of virus assembly and maturation? Is herpesvirus assembly so inefficient to produce these DNA-free particles in very high numbers? Or do these capsid forms represent important intermediates in herpesvirus assembly? These important questions remain unanswered to date due to the inability of present day technology to assess the kinetics of virus structural changes. We employed a drug inhibition assay in order to address these questions. Our results show excessive accumulation of B-capsid forms in infected cells where viral genome encapsidation is blocked indicating that B-capsids could be the intermediate forms that accumulate when the scaffold containing capsids are not allowed to package viral DNA. The primary mechanism of BDCRB function involves inhibition of the formation of unit-length human cytomegalovirus (HCMV) genomes [32,52]. There is a possibility that the BDCRB block may abnormally divert the virus assembly pathway towards the synthesis of abortive capsid forms; however, studies investigating the mechanism of action of BDCRB have shown that BDCRB specifically prevents viral DNA maturation [32]. Interestingly, A-capsids don’t seem to accumulate in BDCRB-blocked cells. If we assume that BDCRB does not impact other stages of herpesvirus assembly, we surmise that the possibility of BDCRB diverting the virus assembly pathway is minimal. Therefore, in the presence of BDCRB, virus assembly continues until the scaffold-containing capsids are formed but does not move beyond that. It is also possible that the observed B-capsids represent a mixture of natural intermediates and abortive particles and A-capsids could be the empty B-capsids, empty C-capsids (the DNA fell out after packaging), or a mixture of the two.

It has been proposed that capsid quality control mechanisms are functional at the inner nuclear membrane, which only allows for the selective budding of genome-containing capsids [53]. Although A- and B-capsids are often seen in the cytoplasm and they are known to undergo successful envelopment and cell egress [11,18], their comparative numbers in the cytoplasm don’t represent the comparative numbers seen in the nucleus, lending support to the theory of selective budding. Lack of progression of B-capsids to the next stage (*i.e.*, C-capsids) would result in accumulation of B-capsids in the nucleoplasm because quality control mechanisms preferentially allow the release of only C-capsids into the cytoplasm [54].

We also found that NI and CI are properly formed in BDCRB-blocked cells; thus genome encapsidation is not a prerequisite for the formation of these compartments. The tegument protein-containing dense bodies dominate the CI in BDCRB-blocked cells. These dense bodies are enveloped and egress from the cells much like infectious virions [18], suggesting that envelopment and cellular egress pathways remain intact even when genome encapsidation is blocked.

Comparison of the limited resolution cryo-EM structure of A- and B-capsids indicates that there are no obvious structural differences between these two capsid types that would account for putative structural instability or inability of these capsids to egress the nucleus (Figure 6). Moreover, these capsid forms are very similar to the virion capsid structure, except for the absence of capsid-associating tegument proteins [51]. These tegument proteins, suggested to be pp150 and pUL48 [51], are believed to impart stability to nucleocapsids during trafficking in the cells [17]. p150 is acquired by nucleocapsids in the host cell nucleus [55], whereas pUL48 is believed to be acquired in the cytoplasm [51]. pp150 associates with A-, B- and C-capsids (R. Tandon, unpublished data), excluding any role of this protein in the generation of C-capsid selectivity. Homologs of HSV UL17 and UL25 form a complex at the vertices of A-, B-, and C-capsids [53] and this CVSC may be involved in the selection of capsids for nuclear egress [53]; but it is not understood how it would select specifically for C-capsids. In summary, A, B, and C-capsids are structurally similar to a degree that suggests selection for nuclear export is a very subtle feature of the capsid, and possibly located at the unique portal vertex.

It has been proposed that generation of defective genome-containing or genome-less virus particles may be important for diverting or overwhelming the innate immune response. This theory doesn’t explain the generation of A- and B-capsids because a seemingly effective quality control mechanism at the nuclear membrane restricts the maturation and envelopment of a majority of these capsids types. Instead, dense bodies, which are highly immunogenic [56], may act to overwhelm the host immune response.

Thus, our data shows accumulation of mainly B-capsids in the infected cell nucleus when viral genome packaging is blocked. Cryo-EM structures of A- and B-capsid forms indicated that these capsid-forms are nearly identical to the mature nucleocapsids; and thus cannot be interpreted as abortive forms for structural reasons. In summary, our data support the notion of B-capsids as intermediate capsid forms rather than as extraneous or abortive capsids.
